# Unraveling Peritoneal Mesothelioma: A Case-Based Discussion on Diagnosis and Management

**DOI:** 10.7759/cureus.94653

**Published:** 2025-10-15

**Authors:** Othmane Zouiten, Latifa Azarou, Hasnaa Hadiri, Hanane Rais, Rhizlane Belbaraka

**Affiliations:** 1 Department of Medical Oncology, Faculty of Medicine and Pharmacy, Mohammed VI University Hospital of Marrakech, Cadi Ayyad University, Marrakech, MAR; 2 Department of Pathology, Mohammed VI University Hospital of Marrakech, Cadi Ayyad University, Marrakech, MAR; 3 Department of Medical Oncology, University Hospital of Marrakesh, Marrakesh, MAR

**Keywords:** cytoreductive surgery, epithelioid subtype, immunohistochemistry (ihc), peritoneal mesothelioma, systemic chemotherapy

## Abstract

Peritoneal mesothelioma is a rare and aggressive tumor, and its management remains poorly standardized in the literature. This case highlights the diagnostic and therapeutic challenges through a 56-year-old woman with no significant medical history or known asbestos exposure, who presented with progressive abdominal distension and postprandial vomiting. Imaging revealed diffuse nodular peritoneal thickening and large-volume ascites. A laparoscopic biopsy confirmed malignant epithelioid mesothelioma, supported by immunohistochemical positivity for cytokeratin 7 (CK7), calretinin, and Wilms tumor 1 (WT1). Treatment was based on systemic chemotherapy with platinum (cisplatin/carboplatin) and pemetrexed, which elicited a rapid initial response. However, the long-term efficacy remains uncertain due to the aggressive nature of the disease. The absence of standardized treatment underscores the necessity for multidisciplinary management.

## Introduction

Peritoneal mesothelioma, representing <1% of all tumors and 15% of mesothelioma cases, is an aggressive malignancy with poor prognosis (20% five-year survival) [1]. While histologically similar to pleural mesothelioma with epithelioid, sarcomatoid, and biphasic subtypes (epithelioid having better outcomes) [2], it differs epidemiologically by affecting younger patients and showing a weaker asbestos association. Emerging research implicates breast cancer gene 1 and 2 (BRCA1/2) mutations and anaplastic lymphoma kinase (ALK) fusions in pathogenesis [3]. These mutations are not just biological curiosities; they are clinically significant because they can be targeted with existing drugs, paving the way for more effective, personalized treatment. This case report presents a female patient with diffuse peritoneal mesothelioma, highlighting diagnostic challenges through clinical, pathological, and molecular analysis while discussing current therapeutic uncertainties, as management remains largely extrapolated from pleural mesothelioma protocols despite distinct clinical behavior.

## Case presentation

We report the case of a 56-year-old woman with no significant medical history, from a rural area, who presented with a one-year history of progressive abdominal distension and postprandial vomiting. These symptoms developed in the context of unquantified weight loss and declining general health. Initial clinical suspicion pointed toward a malignant process due to the refractory nature of her ascites and progressive deterioration.

**Figure 1:**
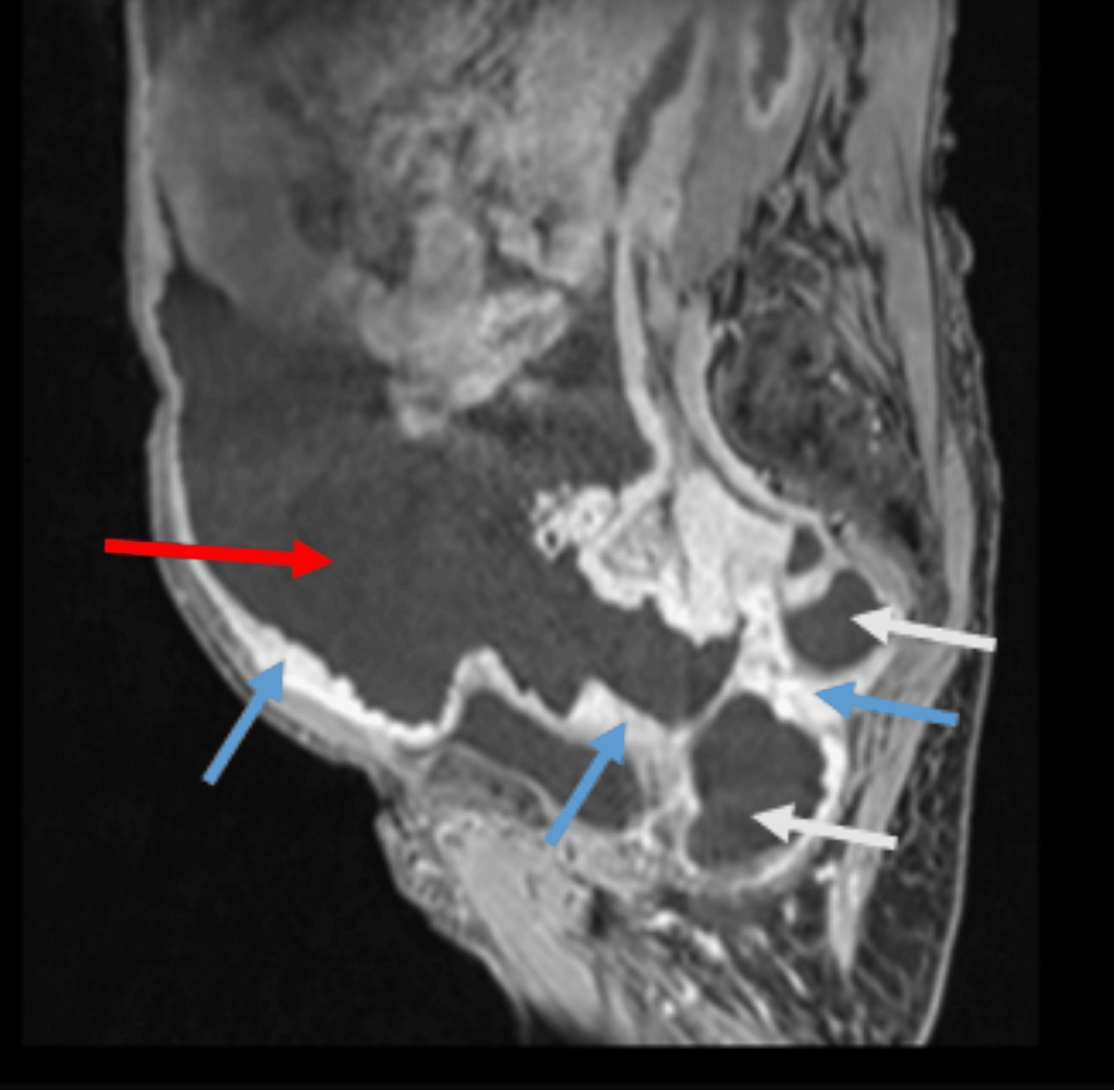
Abdominopelvic MRI revealing characteristic features of peritoneal mesothelioma Coronal MRI image demonstrates large-volume ascites (red arrow) occupying the entire peritoneal cavity. Note the diffuse, nodular peritoneal thickening (blue arrows) and cystic components (white arrows), particularly along the parietal peritoneum. This pattern of diffuse peritoneal involvement, in the absence of a distinct visceral primary tumor, is highly suggestive of peritoneal mesothelioma and helps distinguish it from localized gastrointestinal cancers or the more focal spread of some ovarian malignancies.

An abdominopelvic MRI revealed a large volume of ascites occupying the entire peritoneal cavity, accompanied by diffuse nodular and cystic peritoneal thickening. The maximal thickness measured 28 mm at the left posterior parietal peritoneum and 14 mm at the anterior parietal peritoneum in the hypogastric region. The tumor encased the stomach, colon, mesentery, and small bowel, with the most pronounced wall thickening (18 mm) observed at the left colon. Radiologically, these findings were highly suggestive of peritoneal mesothelioma (Figure 1).

This radiological pattern, characterized by diffuse peritoneal involvement with relative sparing of the visceral organs, is a key feature that helps distinguish peritoneal mesothelioma from more common malignancies. For instance, peritoneal carcinomatosis due to gastrointestinal cancers is often associated with more suggestive symptoms such as gastrointestinal bleeding, while advanced ovarian cancer typically presents with elevated cancer antigen 125 (CA-125) and dominant pelvic masses, which were absent here.

Tumor marker assessment showed low levels of CA-125 at <4 U/mL (normal range: <25 U/mL), cancer antigen 19-9 (CA19-9) of 4.48 U/mL (normal range: <37 U/mL), carcinoembryonic antigen (CEA) of 0.76 ng/mL (normal range: <5 ng/mL), and alpha-fetoprotein (AFP) of 2.91 ng/mL (normal range: <10 ng/mL). This unremarkable profile effectively ruled against a primary gastrointestinal or ovarian malignancy, which typically elevates one or more of these markers.

**Figure 2:**
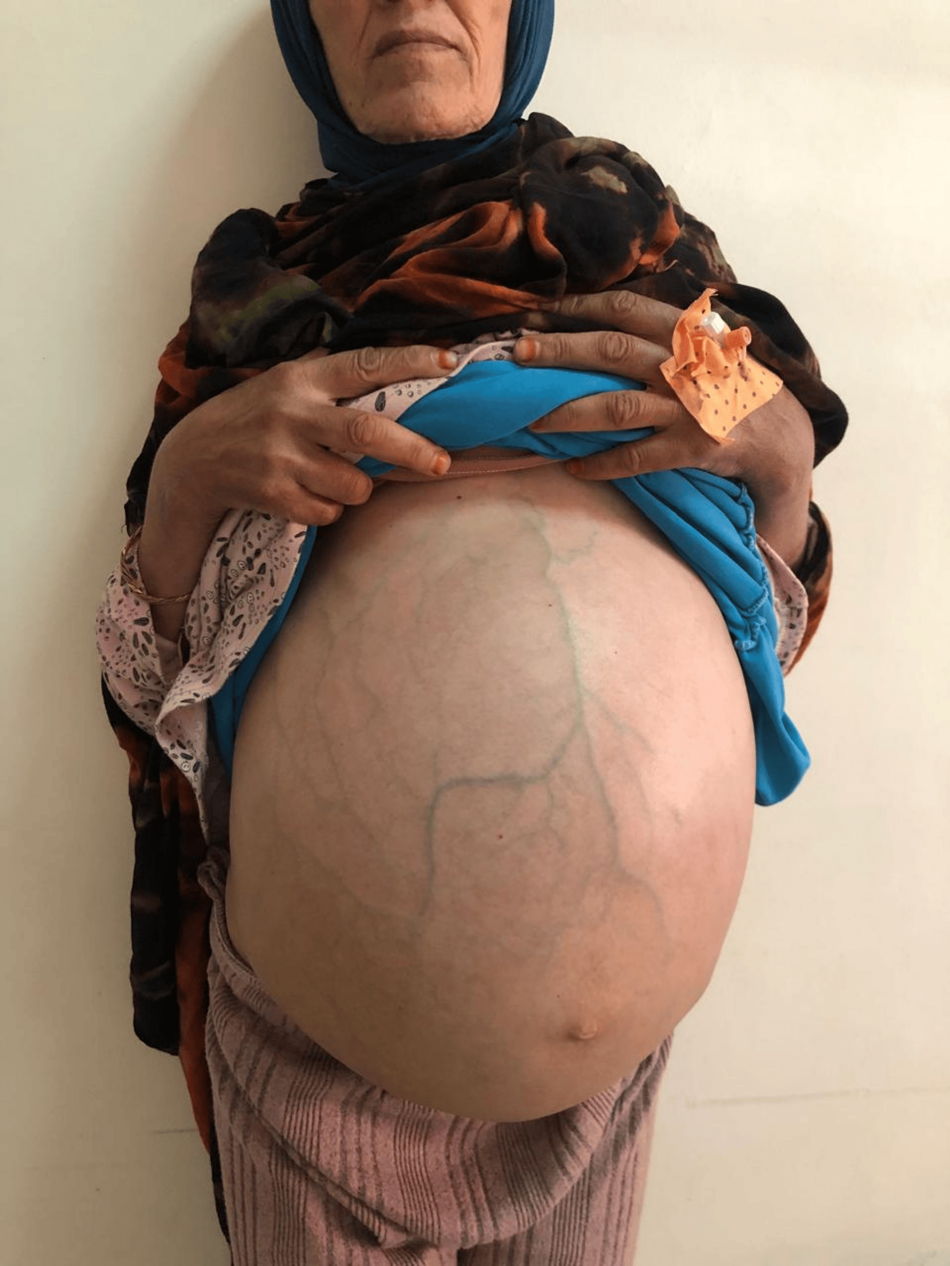
Photograph of the patient's abdomen showing significant distension and bulging flanks, consistent with a large volume of ascites This clinical sign, while nonspecific, in the context of a subacute presentation, should raise suspicion for a malignant process such as peritoneal carcinomatosis, for which mesothelioma is a key differential diagnosis.

Upon referral to medical oncology, clinical examination revealed a patient with no exposure to asbestos and in relatively good general condition, although with significant abdominal distension and shifting dullness consistent with large ascites. Gynecologic and systemic examinations were otherwise unremarkable (Figure 2).

**Figure 3:**
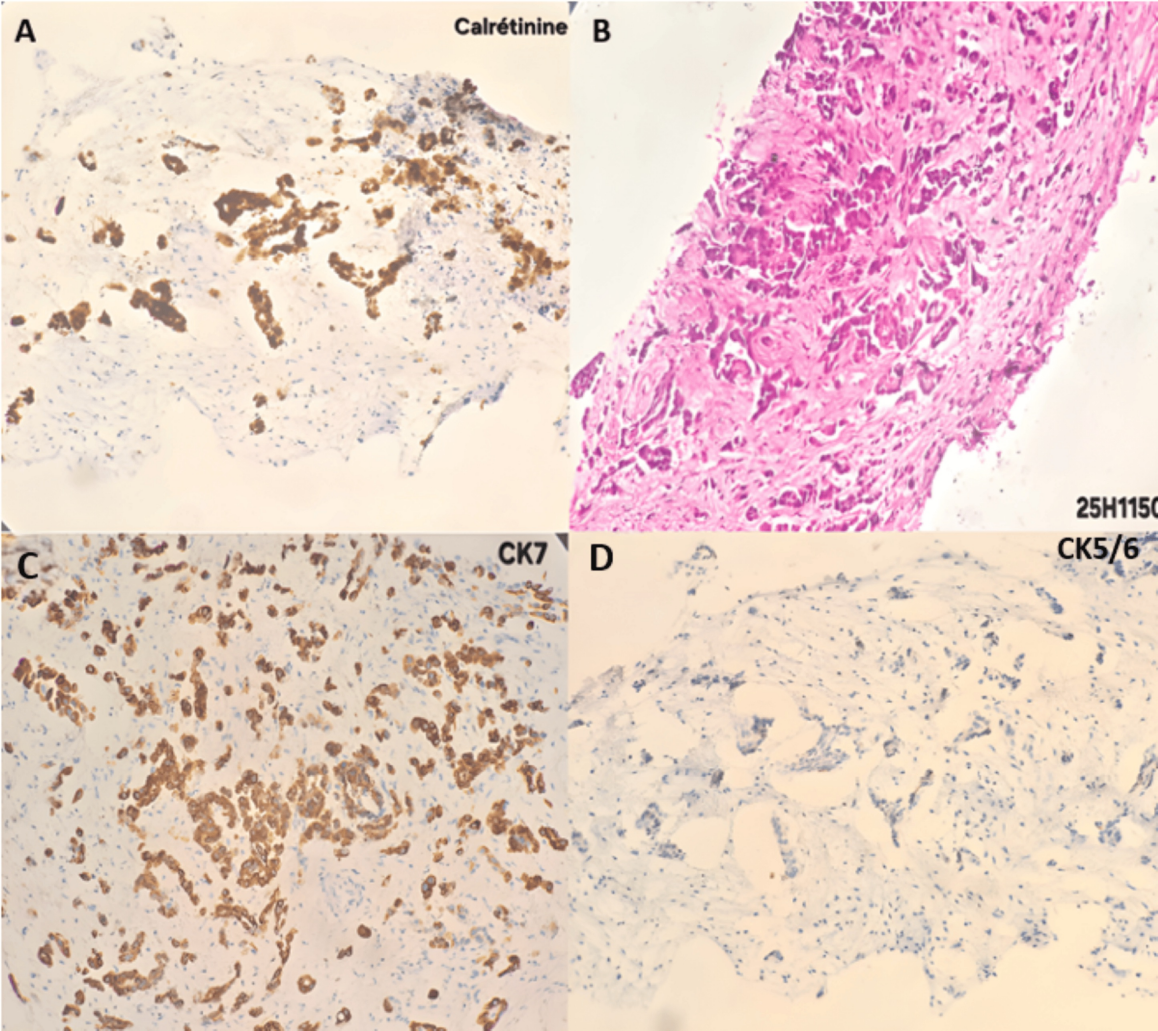
Diagnostic immunohistochemical profile confirming epithelioid mesothelioma Histopathological analysis of the laparoscopic biopsy specimen showing (A) moderate to intense nuclear and cytoplasmic expression of calretinin, (B) moderate nuclear and cytoplasmic expression of P40, (C) intense cytoplasmic expression of CK7, and (D) weak and focal cytoplasmic expression of CK5/6. This immunophenotype (calretinin+/P40+/CK7+) is highly characteristic of epithelioid mesothelioma. Its clinical relevance lies in its ability to definitively exclude other mimics: serous ovarian carcinoma (typically calretinin negative and PAX8 positive) and pulmonary adenocarcinoma (typically TTF-1 positive and calretinin negative), thereby confirming the primary peritoneal origin. CK7: cytokeratin 7, CK5/6: cytokeratin 5/6, PAX8: paired box protein 8, TTF-1: thyroid transcription factor-1

A definitive diagnosis was obtained via laparoscopic biopsy, which demonstrated fibro-adipose tissue infiltrated by a round-cell tumor proliferation. Immunohistochemical analysis confirmed strong positivity for cytokeratin 7 (CK7), calretinin, Wilms tumor 1 (WT1), and CK5/6, while markers such as CK20, thyroid transcription factor-1 (TTF-1), and GATA binding protein 3 (GATA3) were negative. This immunoprofile is diagnostic for epithelioid mesothelioma and is crucial for excluding other mimics, such as serous papillary carcinoma of the peritoneum or ovary (typically WT1 positive but calretinin negative) or metastatic adenocarcinoma (typically CK20 and/or CEA positive). These findings confirmed the diagnosis of malignant epithelioid mesothelioma (Figure 3).

**Figure 4:**
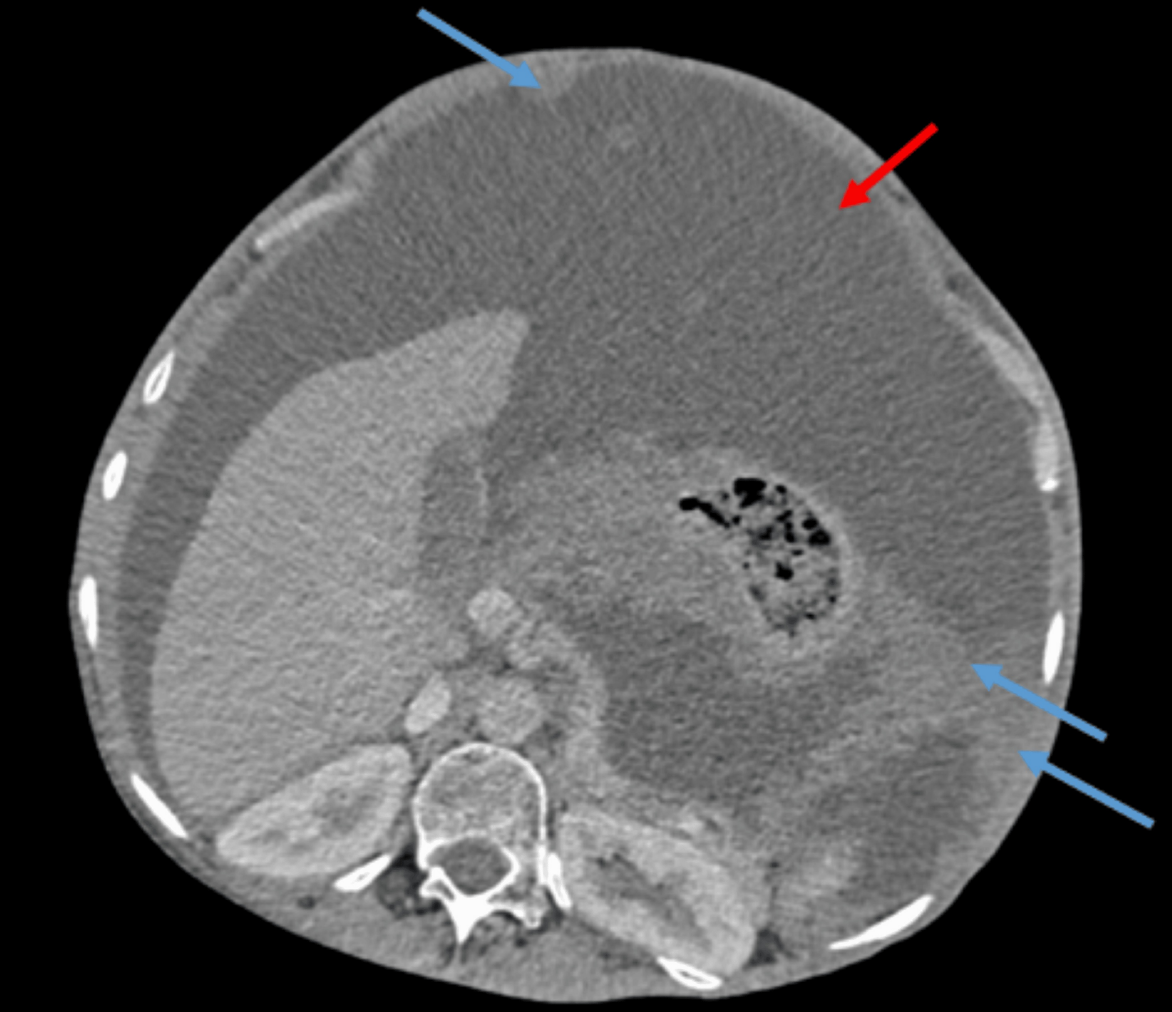
Axial view from a thoraco-abdominopelvic CT scan showing pronounced left-sided peritoneal thickening (blue arrows) and massive ascites (red arrow) This distribution supports the decision for a peritoneal-directed treatment strategy.

Further staging with a thoraco-abdominopelvic CT scan identified a cystic mass in the pouch of Douglas and massive ascites, with no evidence of pulmonary involvement. A multidisciplinary tumor board review deemed the disease unresectable, recommending systemic therapy as the primary treatment approach (Figure 4).

The patient was started on a chemotherapy regimen consisting of cisplatin, pemetrexed, and bevacizumab. After two cycles, she exhibited good clinical tolerance with notable improvement in abdominal symptoms. Treatment adherence was satisfactory, and a follow-up clinical and radiological evaluation is planned after the third cycle to assess therapeutic response.

## Discussion

Peritoneal mesothelioma is a rare malignancy (1% of all tumors and 15% of mesothelioma cases) with distinct clinicopathological features. While pleural mesothelioma predominates in older men with asbestos exposure, peritoneal cases show equal sex distribution and occur in younger patients, including those without known exposure, as exemplified by our 53-year-old Moroccan female patient [1]. Global incidence varies, with underreported cases in regions like South Africa (environmental asbestos) and Morocco (occupational exposure) [4,5]. Symptoms are nonspecific (abdominal distension, pain, and weight loss), often delaying diagnosis (~4 months). Imaging (CT/MRI) reveals peritoneal thickening and ascites, although differentiation from ovarian cancer remains challenging, as seen in our case, where MRI suggested mesothelioma despite initial suspicion of gynecologic malignancy [6]. This diagnostic ambiguity underscores a key limitation of conventional imaging: its reliance on anatomical changes that are often nonspecific. Future research should focus on advanced techniques such as diffusion-weighted MRI or radiomic analysis of texture features to improve diagnostic specificity non-invasively.

Diagnosis hinges on laparoscopy (96.9% accuracy) with histopathology and immunohistochemistry (calretinin+, WT1+, and TTF1-) [2]. While laparoscopy is the gold standard, it is an invasive procedure with inherent risks of morbidity, and its diagnostic yield is contingent upon sampling the correct lesion, representing a significant practical limitation. Our case confirmed the epithelioid subtype via laparoscopic biopsy, effectively excluding adenocarcinoma and guiding subsequent therapy. Molecular profiling identifies critical alterations (BRCA1-associated protein-1 (BAP1) and ALK fusions) and immune markers (programmed death-ligand 1 (PD-L1)), although accessibility limits routine use. Expanding on this, the potential of molecular profiling for personalized treatment is profound. For instance, detecting an ALK fusion not only confirms a diagnosis but also directly mandates a shift from conventional chemotherapy to targeted ALK inhibitors, which can yield dramatic and durable responses [3]. Similarly, PD-L1 expression can stratify patients for immunotherapy, optimizing resource use. Emerging techniques such as PET/CT (86% sensitivity) improve staging but face financial barriers in resource-limited settings [6].

Cytoreductive surgery with hyperthermic intraperitoneal chemotherapy (HIPEC) is the gold standard for resectable disease (five-year survival: 39%-91%), although patient selection is critical (Peritoneal Cancer Index ≤ 17, epithelioid histology) [7]. For unresectable cases, cisplatin-pemetrexed (±gemcitabine) offers modest survival benefits (median overall survival (OS): 8.7-26.8 months) [8]. Our patient responded well to cisplatin-pemetrexed despite inoperability, highlighting the role of systemic therapy. Immunotherapy (e.g., nivolumab-ipilimumab) shows promise (response rate: 19%, median OS: 19 months), particularly in PD-L1+ tumors [9]. This evolving treatment landscape makes a compelling case for the systematic integration of molecular testing into the initial diagnostic workflow to unlock these novel, potentially more effective therapeutic avenues.

Outcomes and prognosis depend on histology (epithelioid favorable), Peritoneal Cancer Index, and nodal status. HIPEC and younger age correlate with improved survival, while sarcomatoid histology or high Ki-67 portend a worse prognosis [10]. Despite advances, therapeutic options remain limited. Our case illustrates the diagnostic pitfalls and potential of chemotherapy in resource-constrained settings. To address these challenges, future efforts must not only advocate for broader access to multimodal therapies but also prioritize the development of less invasive diagnostic tools and the standardization of molecular profiling. This will enable truly personalized treatment protocols, moving beyond histology alone to allocate therapy based on the unique genetic and immunologic signature of each patient's tumor, thereby improving overall survival and quality of life.

## Conclusions

Peritoneal mesothelioma is a challenging malignancy that should be considered in any patient with unexplained ascites, irrespective of asbestos exposure history. Accurate diagnosis relies on immunohistochemical confirmation to distinguish it from other abdominal malignancies. This case demonstrates that systemic platinum-based chemotherapy can provide significant clinical benefit and serve as an important treatment option, particularly in settings where more aggressive surgeries are not feasible. Prompt initiation of therapy is warranted, and molecular profiling may help identify potential targeted therapies for appropriate patients. Looking ahead, future research should focus on validating non-invasive diagnostic biomarkers to reduce delays, expanding access to comprehensive molecular profiling to unlock personalized treatment strategies, and establishing the efficacy of novel agents, including immunotherapy, through dedicated clinical trials. These efforts are crucial to improving survival and quality of life for patients with this rare disease.
